# 1507. The Yield and Correlates of Mandated Annual Screening for Chlamydia Trachomatis and Neisseria Gonorrhea in People Living With HIV in a Large Federally-Funded Clinical Cohort

**DOI:** 10.1093/ofid/ofad500.1342

**Published:** 2023-11-27

**Authors:** Madhu Suryadevara, Eliahu Bishburg, Dennis Hogan

**Affiliations:** RWJBH - Newark Beth Israel Medical Center, Newark, New Jersey; Newark Beth Israel Medical Center, Newark, New Jersey; RWJBH NEWARK BETH ISRAEL MEDICAL CENTER, Princeton, New Jersey

## Abstract

**Background:**

Gonorrhea (GC) and Chlamydia (CT) screening is part of the requirement for grantees of the Ryan White funding of HIV clinics. Tests are required to be done at least once yearly. Testing is required to all patients regardless of age or sexual activity.

Our aim was to assess the yield of GC/CT screening in patients living with HIV (PLWH) in an inner-city HIV clinic.

**Methods:**

Retrospective Chart Review over a 10-year period (2013-2023). Data was collected for demographics, sexual history, HIV Viral Load (VL), CD4 count, syphilis serology, GC/CT urine probe results using nucleic acid amplification testing (Amplicor PCR and Aptima TMA), treatment when infection was diagnosed and outcome.

**Results:**

1120 unique patients were identified, a total of 5050 GC/CT tests were analyzed. Median age of GC/CT (-) pts. was 53.6 yrs. with a range of 21-90 yrs. Positive GC/CT tests was found in 43 pts of whom 28/43 (65%) males, 42/43 (97.6%) were African American. These 43 patients had 56 tests with a positivity rate of 56/5050 (0.01%). Median age of GC/CT (+) pts. was 33.7 yrs. Age range of positive pts. was 21-65 yrs. GC (+) only tests in 13/56 (23%), CT (+) only tests in 43/56 (76.7%). Both GC (+) and CT (+) co-infection in 3/43 (6.9%). Positive syphilis serology concomitant with GC/ CT(+) in 5/43 (11.6%). In the GC/CT (+) group, median VL 43.5 copies/mL and median CD4 534.5 cells µL. 30/43 pts. had undetectable VL. All GC+/CT+ pts. were successfully treated with post treatment negative test documented in all but 1 non adherent patient. All patients were sexually active. 7/43 with prior Sexually Transmitted Disease (STD).

**Conclusion:**

Yield of screening for GC/CT in our clinic was very low. Younger patients had higher positivity rates compared to the elderly. Routine screening for these infections should be targeted at younger patient who are sexually active and/or symptomatic.

**Disclosures:**

**All Authors**: No reported disclosures

